# Co-electrolysis of seawater and carbon dioxide inside a microfluidic reactor to synthesize speciality organics

**DOI:** 10.1038/s41598-023-34456-6

**Published:** 2023-06-26

**Authors:** Saptak Rarotra, Amit Kumar Singh, Tapas Kumar Mandal, Dipankar Bandyopadhyay

**Affiliations:** 1 Department of Chemical Engineering, Indian Institute of Technology Guwahati, Guwahati, Assam 781039 India; 2 Centre for Nanotechnology, Indian Institute of Technology Guwahati, Guwahati, Assam 781039 India; 3 Present Address: Energy Research Institute, Nanyang Technological University, Singapore, 637553 Singapore; 4 Present Address: Department of Mechanical Engineering, George Mason University, Fairfax, VA 22030 USA

**Keywords:** Devices for energy harvesting, Chemical engineering

## Abstract

We report co-electrolysis of seawater and carbon dioxide (CO_2_) gas in a solar cell-integrated membraneless microfluidic reactor for continuous synthesis of organic products. The microfluidic reactor was fabricated using polydimethylsiloxane substrate comprising of a central microchannel with a pair of inlets for injection of CO_2_ gas and seawater and an outlet for removal of organic products. A pair of copper electrodes were inserted into microchannel to ensure its direct interaction with incoming CO_2_ gas and seawater as they pass into the microchannel. The coupling of solar cell panels with electrodes generated a high-intensity electrical field across the electrodes at low voltage, which facilitated the co-electrolysis of CO_2_ and seawater. The paired electrolysis of CO_2_ gas and seawater produced a range of industrially important organics under influence of solar cell-mediated external electric field. The, as synthesized, organic compounds were collected downstream and identified using characterization techniques. Furthermore, the probable underlying electrochemical reaction mechanisms near the electrodes were proposed for synthesis of organic products. The inclusion of greenhouse CO_2_ gas as reactant, seawater as electrolyte, and solar energy as an inexpensive electric source for co-electrolysis initiation makes the microreactor a low-cost and sustainable alternative for CO_2_ sequestration and synthesis of organic compounds.

## Introduction

The microreactor consists of continuous-flow microchannels with a typical diameter of under 1 mm and reaction volumes ranging from nanolitres to microlitres^1–3^. Over the last decade, microreactors have revolutionized pharmaceutical industries^4–6^, point-of-care diagnostics^7^, clean energy^8,9^, and high-throughput chemical synthesis^10–12^. Compared to batch processes, microreactor technology allows continuous synthesis of commercial products with reduced reaction times, improved yield, improved selectivity, higher efficiency, greater profitability, precise reaction control, waste reduction, and safe handling of hazardous reactions^13,14^. Microreactors can be substantially used for CO_2_ gas utilization and sequestration in order to synthesize organic compounds^15–17^.

The high levels of CO_2_ emissions from industrial activities, automobile exhausts, or fossil fuel combustion have triggered unwanted climate change, global warming, and severe environmental stress around the world^18^. As a result, innovative solutions are needed to reduce CO_2_ emissions and convert CO_2_ gas into commercial products^19^. In practice, CO_2_ capture, extraction, purification, and conversion by chemical or electrochemical processes are currently hindered by their high cost and energy requirements^20^. The most promising approach for CO_2_ sequestration is to convert the pristine CO_2_ gas into products such as alcohol, carboxylic acid, aldehyde, ester, ketone, paraffin, or solvent for industrial use^21,22^. Previous studies have shown that the macroscopic batch reactors can be employed for electrochemical reduction of atmospheric CO_2_ into methanol^23–25^. In recent past, aside from processing oil, diesel or other liquid hydrocarbon mixtures, CO_2_ from the flue gases has subjected to electrochemical reduction in order to produce a variety of value-added organic products, such as formic acid (HCOOH), formaldehyde (HCHO), and methanol (CH_3_OH)^26–29^. Furthermore, the catalyst-assisted reverse water–gas-shift reaction systems have also been developed for conversion of atmospheric CO_2_ into hydrocarbon fuels in presence of seawater^30^. Recent studies have demonstrated that an electrochemical cell can produce carboxylic acid, glycol, and carboxylate compounds by simultaneously electrolyzing H_2_O in one compartment while reducing CO_2_ in another compartment^31–35^. It has been demonstrated that co-electrolysis of CO_2_ and H_2_O with alternative energy sources such as wind and solar irradiation can be used to produce hydrocarbon fuels and industrial chemicals^36–38^. Literature reports have shown that microchannel reactors can reduce CO_2_ under galvanic conditions by catalytic electrochemical reduction^39,40^. An efficient conversion of greenhouse CO_2_ gas into value-added hydrocarbons would be feasible with the development of a catalyst-free solar-powered microreactor system.

Herein, we report a portable, self-reliant, and low-cost polydimethylsiloxane (PDMS) microreactor for continuously producing a variety of commercially significant organic compounds in a sustainable manner using naturally abundant CO_2_, seawater, and solar energy. A proof-of-concept membraneless microreactor for CO_2_ utilisation is developed with the following components—(i) an integrated T-shaped microchannel with a photovoltaic cell and conductive contacts to produce a high-intensity electrical field inside the microchannel which will allow CO_2_ to be chemically converted under solar radiation, (ii) two inlets—one for incoming CO_2_ gas and one for the inflow of seawater, and an outlet for collecting organic compounds. Since the integrated solar panels convert solar illumination into electrical energy, the proposed microreactor requires low operating electrical power for chemical reactions. The embedded electrodes, separated by a microscale gap, generate a high intensity field within the microchannel reactor at lower potential differences. When CO_2_ and seawater are introduced into the microreactor, the gas-liquid mixture encounters the high intensity electrical field inside the microreactor, and undergoes rapid co-electrolysis to produce nascent hydrogen (H_2_) and oxygen (O_2_) as well as free radicals or ions. By using solar illumination, a variety of organic compounds can be produced, including aldehydes, formate salts, formic acid, primary and secondary alcohols, and hydrocarbons. The microreactor mimics plant photosynthesis by converting CO_2_ gas and water into organic products^41^. The novelty of the work lies in the fact that it is arguably the first research in which a catalyst-free PV cell-integrated microreactor has been employed to transform solar energy into electricity and then, with the help of generated electricity, a gas–liquid mixture of CO_2_ and seawater has been turned into an array of organic products. Importantly, further investigations suggest that the reaction rate and the organic compound to be synthesized inside the microreactor can be tuned by regulating the electric field strength produced by the PV cells under solar irradiation.

In addition, Fourier-transform infrared (FT-IR) spectroscopy, gas chromatography-mass spectrometry (GC-MS), and high-performance liquid chromatography (HPLC) methods were used to analyse and categorize the organic products obtained from the microreactor, and to understand how these organic products are formed by high intensity electrical fields within the reactor. The assembly of these microreactor prototypes can be used to intensify traditional CO_2_ utilization processes in the near future due to their high energy efficiency and low cost of operation. In brief, the proposed microreactor opens up a promising avenue for CO_2_ sequestration and clean energy, which may have far-reaching implications for the mitigating global warming.

## Results

**Figure 1:**
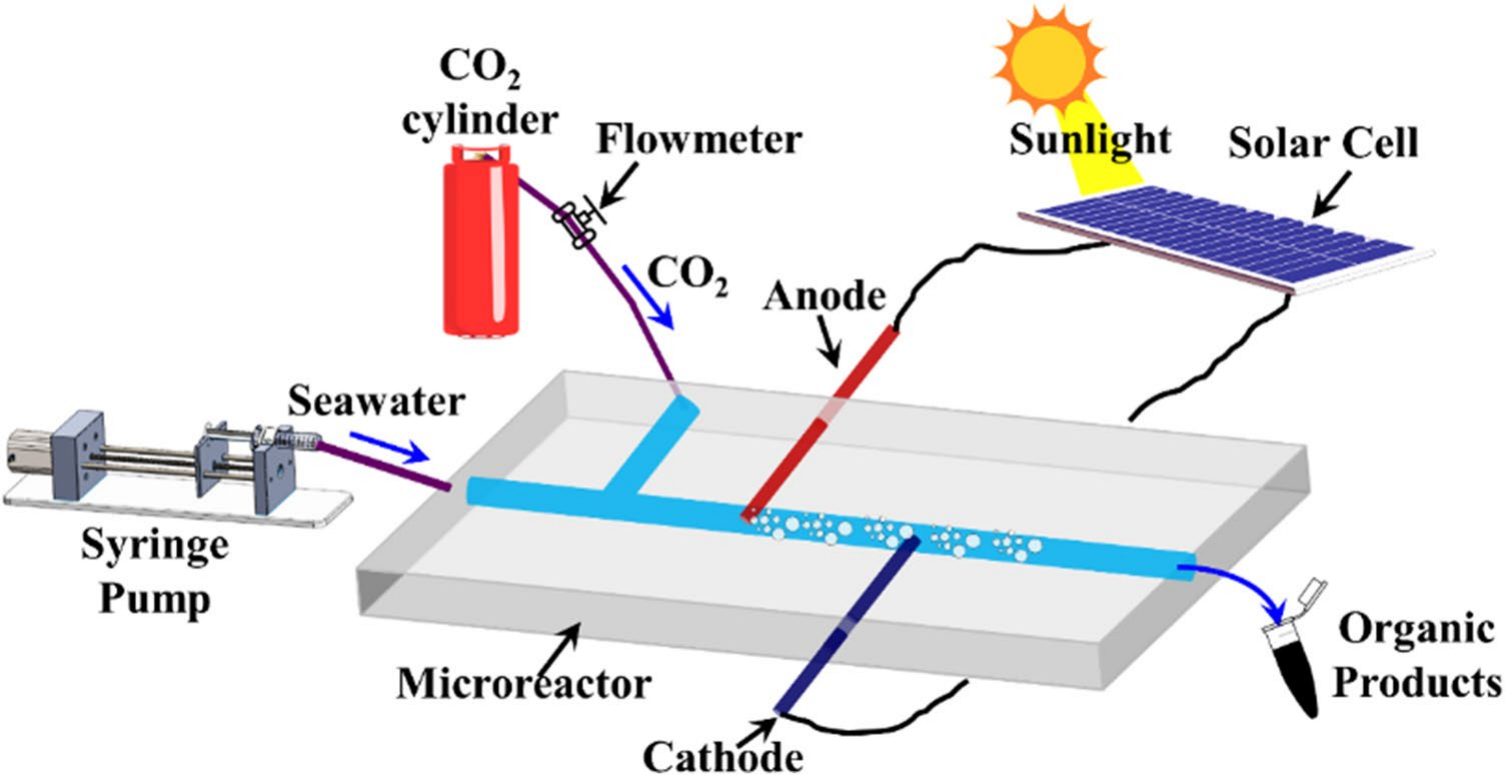
Schematic illustration of the experimental set-up showing microreactor for CO_2_-sequestration. The microreactor has two inlets perpendicular to each other, one for seawater connected to the syringe pump operating at a constant flow-rate (*Q*_*w*_ = 3 mL/min) and another for injection of gaseous CO_2_ from a pure CO_2_ gas cylinder with a mass flow meter operating at a constant flow-rate (*Q*_*g*_ = 3 mL/min). The integrated Cu electrodes are positioned perpendicular to the gas–liquid flow and connected to a solar panel circuitry. The microreactor operates under sunlight and the organic products are collected at downstream of microchannel.

The sequential steps for fabrication process and the dimension details of the membraneless PDMS-based microreactor can be found in Fig. S1 in the electronic supplementary information (ESI). The Fig. 1 shows the experimental set-up of the microreactor employed for the continuous production of diverse organic compounds from greenhouse CO_2_ gas, seawater, and natural sunlight. The microreactor consists of a T-shaped microchannel, two inlets for simultaneous injection of an uninterrupted supply of CO_2_ and seawater along with integrated copper (Cu) electrodes for the generation of an electric field for the chemical processing of CO_2_ under solar irradiation. The mass flow rate of CO_2_ gas (*Q*_*g*_) and seawater (*Q*_*w*_) were maintained at a constant rate of constant rate of 3 mL/min for all the experiments, unless stated otherwise. The two electrodes, positioned several microns apart, were mounted perpendicular to the incoming gas-liquid flow and attached to the outer solar panel to transform solar energy into electrical energy. Under solar irradiation, the closely spaced electrodes produced a strong localized electrical field, thereby, resulting in generation of a high electric potential gradient (*ψ*) within the microchannel. The gas-liquid mixture of CO_2_ and seawater were flown through the microfluidic reactor to produce various organic chemicals such as formate salts, formic acid, primary or secondary alcohols, hydrocarbons along with hydrogen and oxygen under direct solar illumination. Importantly, the rate and composition of as-synthesized organic products were adjusted by regulating the field strength across the electrodes, which, in turn, was controlled by the adjusting the number of solar grid cells exposed to direct sunlight. The electric field potential (*ψ*) inside the microreactor was varied from 2.5 V to 3.5 V for catalyst-free rapid co-electrolysis of seawater and CO_2_ gas and the generated chemical products were collected and analyzed by various characterization techniques.

### Proposed electrode‐reaction mechanisms

#### Reaction at cathode

**Figure 2:**
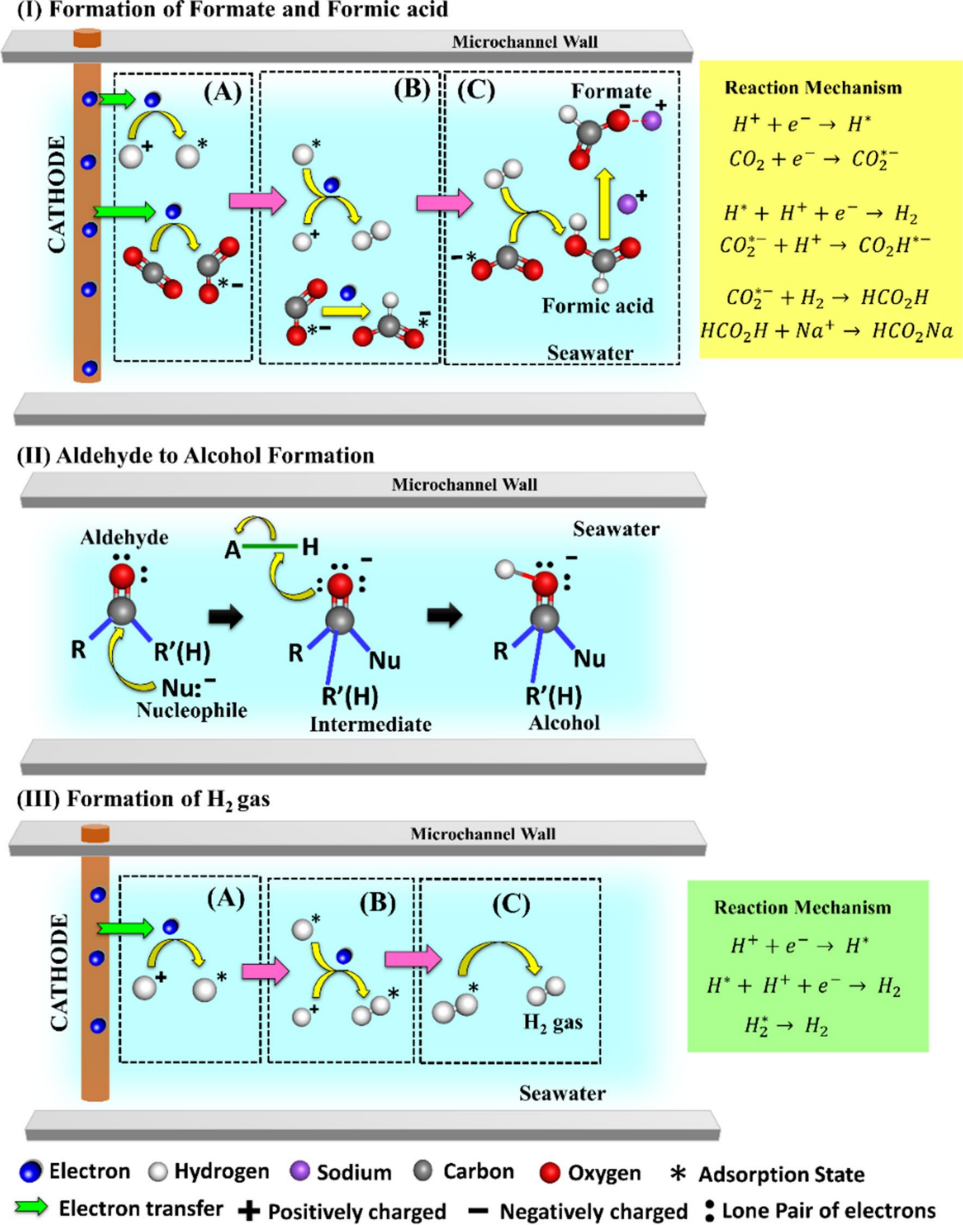
The proposed electrode-reaction mechanism for organic product formation near the cathode of the microreactor. The image (**IA–C**) shows the CO_2_ reduction reaction at cathode leading to the formation of formate and formic acid. The image (**IIA–C**) shows the conversion of to alcohol near cathode, and the image (**IIIA–C**) shows the CO_2_ reduction reaction at cathode resulting in H_2_ gas evolution.

The Fig. 2 shows the proposed electrode mechanism at the cathodic region of the microreactor. The Fig. 2IA–C depicts the reaction mechanisms involved in the formation of the formate (HCO_2_^−^) ions and formic acid (CH_2_O_2_) molecules. The reaction occurs in several steps—(i) gaseous CO_2_ is absorbed (CO_2_^*^) on the cathode surface (Fig. 2IA) and then it undergoes electron (e^-^) addition to yield CO_2_^*−^ species as the electron transfer takes place from cathode to the incoming CO_2_ gas (CO_2 _+ e^− ^→ CO_2_^*−^); (ii) formation of adsorbed formate ion (CO_2_H^*−^) occurs (Fig. 2IB) via protonation (H^+^) of CO_2_^*−^ species (CO_2_^*− ^+ H^+ ^→ CO_2_H^*−^); (c) the CO_2_^*−^ species combines with liberated H_2_ gas to yield formic acid (CO_2_^*− ^+ H_2 _→ CO_2_H_2_) as shown in Fig. 2 IC, furthermore the generated formic acid molecules combines with sodium (Na^+^) ions present in salty seawater to form formate salts (CO_2_H_2 _+ Na^+ ^→ HCO_2_Na). The Fig. 2IIA–C shows the conversion of the aldehydes into alcohols via nucleophilic addition reaction, as aldehydes were more liable to the nucleophilic addition because of their trigonal planar geometry. In such reactions, the nucleophiles used their electron pairs to form a bond with the carbonyl carbon atom. As this happened, the electron pair of carbon–oxygen bond loosened out towards electronegative carbonyl oxygen atom and hybridization state of carbon and oxygen changes from sp^2^ to sp^3^ hybridization. In the second step, the oxygen atom accepts a proton, resulting in conversion of aldehydes to alcohols. The Fig. 2IIIA–C represents the hydrogen evolution reaction, which took place in a series of steps—(i) electron addition (electronation) occurred to subsequent H + ions to yield adsorbed hydrogen (H^*^), as shown in Fig. 2IIIA (H^+ ^+ e^− ^→ H^*^); (ii) the adsorbed hydrogen gas molecule (H_2_^*^) is formed by addition of H^+^ ions (protonation) to the adsorbed hydrogen (H^*^), as shown in Fig. 2IIIB (H^+ ^+ e^− ^+ H^* ^→ H_2_^*^); (iii) conversion and desorption of the adsorbed hydrogen gas molecule (H_2_^*^) took place simultaneously, to form H_2_ gas at the cathode (H_2_^* ^→ H_2_), as depicted in the Fig. 2IIIC.

#### Reaction at anode

**Figure 3:**
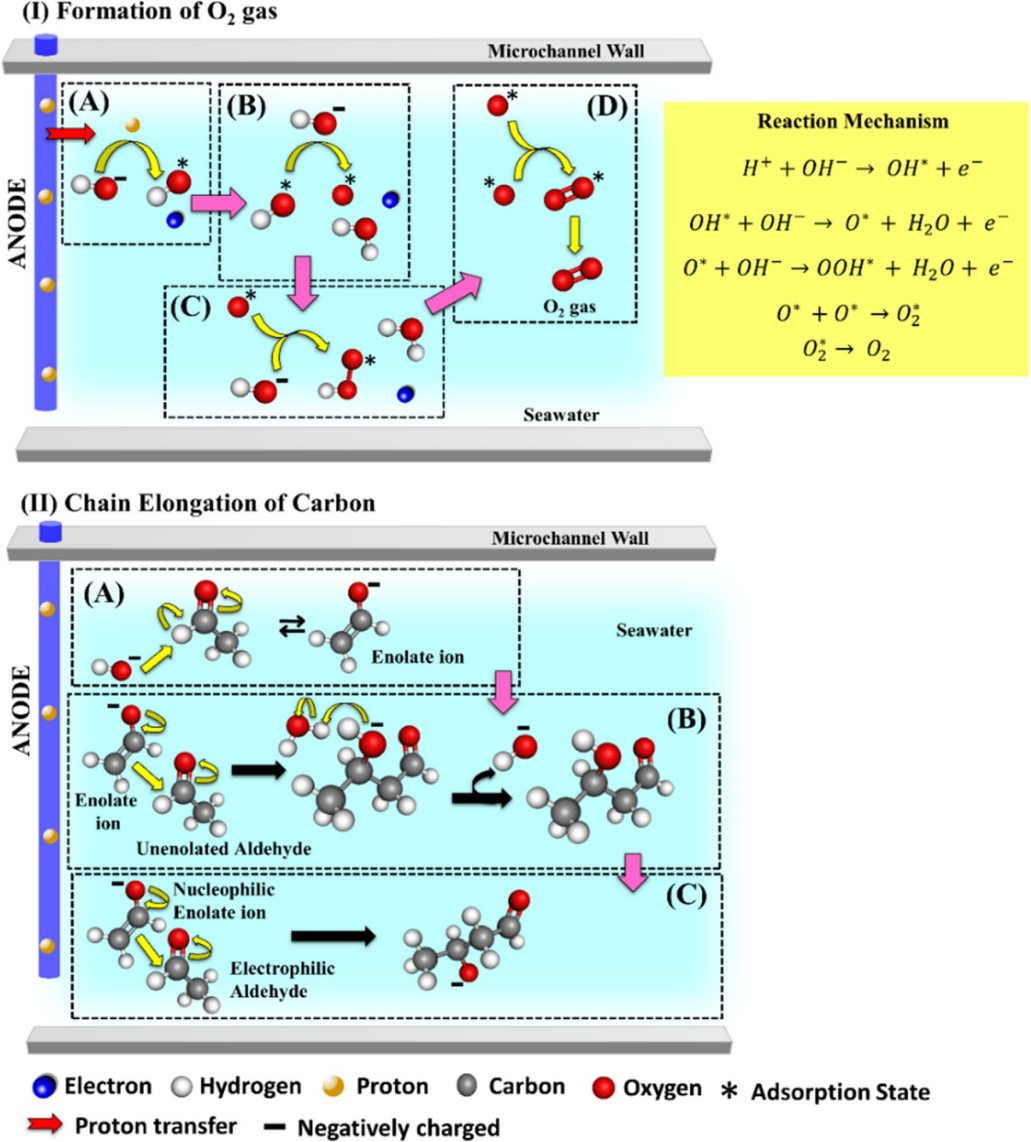
The proposed electrode-reaction mechanism for organic product formation near the anode of the microreactor. The images (IA–D) represent the oxidation reaction at anode in the form of oxygen evolution and the image (IIA–C) shows the carbon-chain elongation reaction near anode.

The Fig. 3 shows the proposed electrode mechanism at the anodic region of the microreactor. The electrode-reaction at the anode assisted the evolution of O_2_ gas along with carbon chain elongation. The Fig. 3IA–D shows the proposed mechanisms for the O_2_ gas evolution—(i) the reaction is initiated by adsorption of free hydroxide (*OH*^*-*^) ion (Fig. 3IA) near the anode (OH^− ^+ H^+ ^→ OH^* ^+ e^−^) ; (ii) the adsorbed hydroxide (OH^*^) gets converted to adsorbed oxygen (O^*^) (Fig. 3IB) via oxidation (OH^* ^+ OH^− ^→ O^* ^+ H_2_O + e^−^) ; (iii) oxygen coupling reaction takes place for the formation of hydroperoxyl (OOH^*^) radical (Fig. 3IC) via oxidation (O^* ^+ OH^− ^→ OOH^* ^+ H_2_O + e^−^); (iv) the conversion and desorption adsorbed oxygen (O^*^) occurs simultaneously to form O_2_ gas at the anode (O_2_^* ^→ O_2_), as shown in Fig. 3ID. Furthermore, the Fig. 3IIA–C shows the proposed mechanism of carbon-chain elongation reaction via following steps—(i) formation of a nucleophilic enolate ion by adding hydroxide ion (OH^−^) to an synthesized as an intermediate product (Fig. 3IIA); (ii) the enolate molecule interacts with the non-enolized molecules, which facilitated the nucleophilic enolate ions to attack one of the non–enolized molecules to form an alkoxide ion (Fig. 3IIB) that was further protonated by H_2_O molecule to form aldol species; and finally (iii) the chain elongation carbon backbone occurs when the nucleophilic enolate attacks the electrophilic aldehyde (Fig. 3IIC).

### FT-IR studies

**Figure 4:**
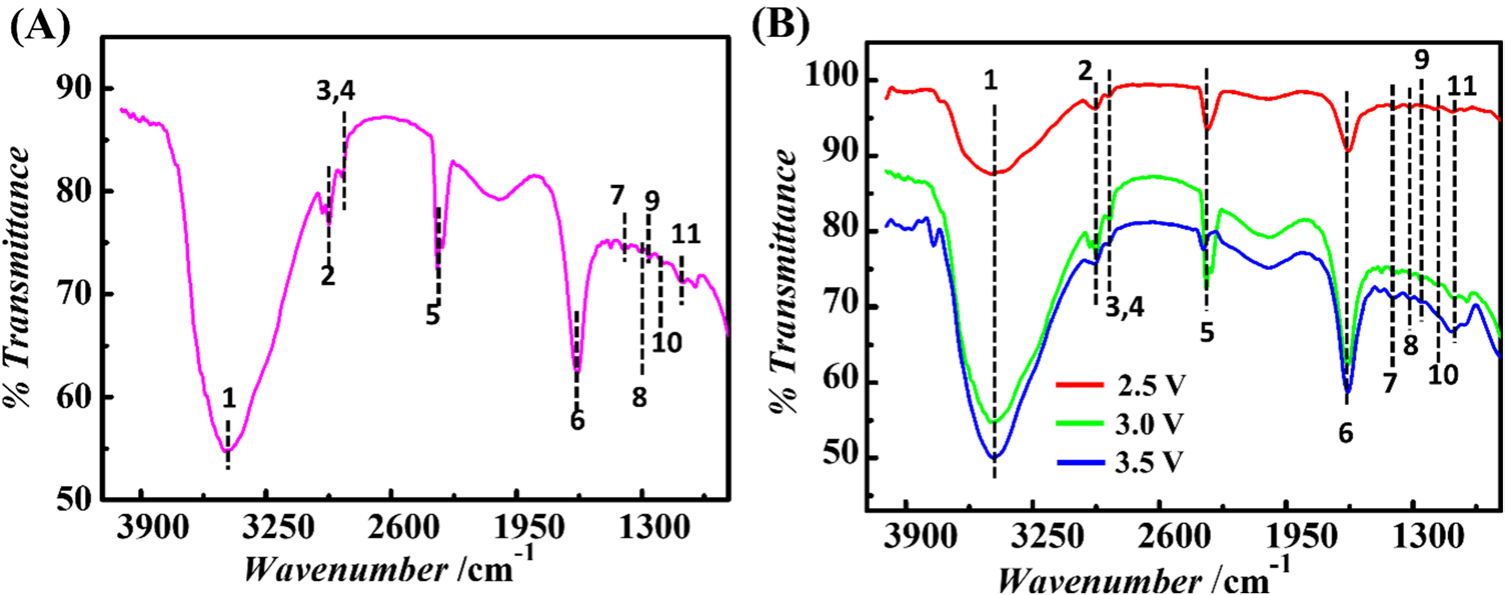
(**A**) The FT-IR spectrum of organic products obtained in aqueous solution from the outlet of the microreactor, operating at applied electric potential (*ψ*) of 3.5 V. (**B**) The normalized FT-IR spectra of organic products obtained from microreactor at applied potential (*ψ*) of 2.5 V (red line), 3.0 V (green line), and 3.5 V (blue line), respectively across the electrodes. A steady flow rate of 3 mL/ min for incoming seawater and CO_2_ streams was maintained for all experiments. The numbers 1–11 depict the assigned functional groups as mentioned in the Table 1.

The Fig. 4A shows the FT-IR spectra of the synthesized organic products ranging from formate salts, formic acid, primary or secondary alcohols, to hydrocarbons, collected downstream from microreactor when an electric potential (*ψ*) of 3.5 V was applied across the electrodes, and a steady flow rate of 3 mL/min for seawater (*Q*_*w*_) and CO_2_ (*Q*_*g*_) was maintained within the microchannel. The aqueous solution collected from the microreactor was vacuum-dried to yield powdery sample for FT-IR analysis. The details of the sample preparation for FT-IR analysis have been mentioned in the ESI. The peaks between 3584 and 3230 cm^−1^ show the formation of alcohols, phenols, and peak at 2924–2859 cm^−1^ suggests the generation of alkanes, ether, aldehydes, while the peak between 1430 and 1400 cm^−1^ depicts the production of esters^42^. In order to study the effect of the electrical energy produced by the solar panel on the synthesis of organic compounds, the electrical potential (*ψ*) across the integrated electrodes inside the microreactor was varied. The Fig. 4B shows the normalized spectra of organic compounds obtained from microreactor at applied electric potential (*ψ*) values of 2.5 V, 3 V, and 3.5 V and at *Q*_*w *_= 3 ml/min and *Q*_*g *_= 3 ml/min, respectively, thus, confirming that the product distribution depends on the applied potential (*ψ*). The recorded FT-IR spectral signatures clearly indicate the formation of an assemble of organic compounds (Table 1) such as formate salts, formic acid, primary or secondary alcohols, and hydrocarbons. The FT-IR spectra confirmed the presence of secondary alcohols, higher esters, primary alcohol, esters of aromatic acids, and non-conjugated alkenes in aqueous product sample^42^.

**Table 1:** The FT-IR analysis was done to identify different organic species present in the aqueous product, collected from the microreactor operating at 2.5–3.0 V.

Assigned Peak no. in Fig. 4	Peak position in Fig. 4 (cm^−1^)	Assigned functional groups	Classification of the Organic compounds likely to present as end product^42^	Vibration mode	Typical vibrational frequency range (cm^−1^)^42^	Peak intensity
1	3454	O–H	Dimeric Alcohols/phenols	Stretching	3584–3230	Strong
2	2922	–CH_2_–	Alkane	Stretching	2924–2859	Strong
3	2859	–O–CH_3_	Ether	Stretching	2924–2859	Strong
4	2859	–CHO	Aldehydes	Stretching	2924–2859	Strong
5	2361	C=NH^+^	NH^+^ Charged amines	Stretching	2500–2325	Weak
6	1632	C=C	Non-conjugated Alkene	Stretching	1635	Strong
7	1401	C–H	Esters	Bending	1430–1400	Weak
8	1314	C–OH	Primary alcohols	Stretching	1350–1260	Weak
9	1257	C–O	Esters of aromatic acids	Stretching	1300–1250	Weak
10	1181	C–O	Higher Esters	Stretching	1200–1170	Weak
11	1100	C–OH	Secondary alcohols	Stretching	1120–1100	Weak

The table shows the classification of the various functional groups based upon the peak positions in the FT-IR spectra reported in Fig. 4

### GC–MS and GC studies

The aqueous end-product comprising of all the reaction intermediates was collected in a closed vial from the microreactor outlet and was subjected to GC-MS analysis. The details of the defined parameters for GC-MS analysis, such as lock peak width, peak width (sec.), slope sensitivity (SN), tangent %, peak size reject (counts), and smoothing parameter, has been mentioned in the Table S1 of ESI.

The chemical species were analyzed and the corresponding data was validated in the NIST-2010 database in order to classify the organic compounds as per their composition. The identified compounds were confirmed by retention time (min.), probable compound name, area %, molecular weight, as summarized in Table 2. The GC-MS confirms the formation of organic products like formate salts, formic acid, primary or secondary alcohols, and hydrocarbons, which correlates with the FT-IR results. For example, the alkenes were detected at retention time of ~ 7.771 min, while the ester were observed at retention time of ~26.514 min in the GC-MS studies, and these two functional groups compounds were also reported in the Fig. 4 and Table 1 of the FT-IR analysis. The generation of the other organic compounds such as secondary alcohols, higher esters, primary alcohol, alkane, and alcohols were verified in the similar manner. The Tables 1 and 2 together confirmed the synthesis of a large number of organic products from the electrochemical reaction between sea water and CO_2_ within the microreactor under the influence of applied electric potential gradient.

**Table 2:** Shows the details of GC–MS for the organic products obtained from microfluidic reactor operating at an electric potential value (*ψ*) ranging from 2.5 V to 3.5 V.

Retention time, *t*_*R*_ (min)	Compound name	Area %	Molecular weight (a.m.u.)
3.443	Butanenitrile,2,3-dioxo-, dioxime, o, o′-diacetyl-	51.33	211
3.5	Cyclopropane,1,2-dimethyl-, trans	25.15	70
	2-Butene, 2-methyl-	16.25	70
5.125	Oxalic acid, diallyl ester	21.36	170
7.771	Spiro [2.4] hepta-4,6-diene	39.48	92
	Cyclobutene, 2-propenylidene	10.95	92
9.937	1-Hexene, 3,5-dimethyl-	6.14	112
	Diphosphoric acid, diisooctyl ester	3.85	402
10.067	Cyclopentane,1,2-dimethyl-3-(1-methylethyl)-	19.89	140
11.211	Cyclohexane, 1,3-dimethyl-2-methylene-, cis-	16.63	124
13.434	Bicyclo [3.1.0] hex-2-ene,2-methyl-5-(1-methylethyl)-	17.70	136
18.796	Hydroxylamine, O-decyl-	5.22	173
24.178	Benzaldehyde, 4-propyl-	46.75	148
26.514	Nonanoic acid, 9-oxo-, methyl ester	59.56	186
29.972	2,3-Dihydroxypropyl cis-13-docosenoate	28.83	412
31.082	Pentadecanoic acid, 14-methyl-, methyl ester	54.99	270
32.545	9-Octadecenoic acid (Z)-, methyl ester	8.48	296
	12-Octadecenoicacid, methyl ester	2.84	296

The gaseous products of the reaction were obtained from the closed vial with the aid of a 100 μL gastight syringe and inserted into the GC TCD port for identification. Two distinct peaks were detected in the GC analysis, one with retention time (*t*_*R*_) of ~ 0.72 min and another with ~ 1.83 min represented H_2_ and O_2_, respectively. Furthermore, in order to quantify the amount of H_2_ and O_2_ gas produced from the reaction, initially, a calibration curve for pure H_2_ gas was obtained using a GC instrument. In the calibration method, different volumes of pure H_2_ and O_2_ gases (0.1–0.9 ml) were collected in 100 μL gastight syringe before injecting into the TCD port of the GC. Thereafter, for a known volume of H_2_ and O_2_ gases, we obtained a typical GC peak in which the *t*_*R*_ was in the range of ~ 0.6–1 min (refer Fig. S2A of ESI) for H_2_ gas and a characteristic GC peak for O_2_ gas with *t*_*R*_ in the range of ~ 1.5–2.5 min (refer Fig. S3A of ESI).

A linear GC calibration plot between the volume of the pure H_2_ gas (*V*_H_) and the area under the curve (*A*_H_) was obtained from this experiment, as shown in the Fig. S2B of the ESI. The Fig. S2B in the ESI shows the linear calibration plot between the volume of pure H_2_ gas (*V*_H_) with the area under the curve (*A*_H_), which led to the correlation *A*_H _= (1.149 × 10^6^)* V*_H _+ 6050.9. Similarly, a linear GC calibration plot between the volume of the pure O_2_ gas (*V*_o_) and the area under the curve (*A*_o_) was obtained from this experiment, as shown in the Fig. S3B of ESI. The calibrated linear correlation for the volume of pure O_2_ (*V*_O_) with the area under the curves (*A*_O_) was obtained as, *A*_O _= (3.2735 × 10^4^) *V*_O _+ 472.8, as shown in the Fig. S3B of the ESI.

**Figure 5:**
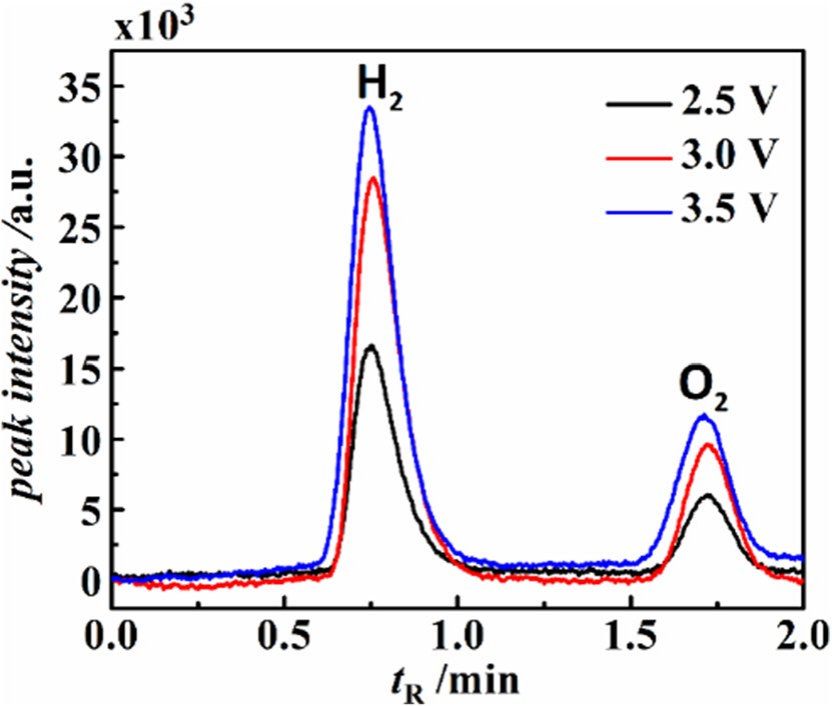
Shows the GC analysis of the gases obtained from the microreactor at applied electric potential (*ψ*) value of 2.5 V, 3.0 V, and 3.5 V. The flow rate of seawater and CO_2_ was maintained at 3 mL/min in all the experiments. The peaks at the retention times of ~ 0.72 min and ~ 1.83 min represented H_2_ and O_2_, respectively. The intensity of the peaks was higher (lower) for hydrogen (oxygen) having a broader (smaller) area under the curves.

The gaseous products issuing out of the microreactor were analyzed in GC under same condition using the correlations obtained from the calibration plot as shown in the Fig. S2 and Fig. S3 of the ESI. Interestingly, the GC plots reported that the mixture of gases collected from the micro-reactor was oxy-hydrogen, which was produced by rapid co-electrolysis of CO_2_ and seawater at very high electrical field strength within the microchannel near the electrode area. The Fig. 5 shows that when the applied potential values (*ψ)* were gradually increased from 2.5 V, 3.0 V to 3.5 V, the peak intensities of H_2_ and O_2_ were also increased. This observation clearly suggested that the rate of electrolysis is increased with the increment in applied voltage, resulting in the rise in volume of evolved H_2_ and O_2_ gases from co-electrolysis of CO_2_ and seawater.

The Fig. 5 shows the GC analysis of the gaseous products collected from the microreactor at the applied electric potential (*ψ*) value of 2.5 V, 3 V, and 3.5 V, where oxy-hydrogen was collected from the outlet of microfluidic reactor^9,43^. The peaks near the *t*_R_ values of ~ 0.72 min and ~ 1.83 min, shown in the Fig. 5 correspond to H_2_ and O_2_ gases, respectively as *t*_R_ of the evolved gases were in consistent with the pure H_2_ and O_2_ gases used for the calibration plot. From the calibration curve (refer Fig. S2B of ESI).

### HPLC studies

The details of the HPLC analysis have been reported in Fig. S4 of the ESI. In the Fig. S4A in ESI, the peaks near the retention time (*t*_R_) value of ~ 2.79 min and ~ 3.52 min. corresponds to formate and formic acid obtained from the microreactor, which further validates the FT-IR and GC-MS analysis results. The Fig. S4B (refer to ESI) shows the linear correlation between the volume of pure formic acid (*V*_FA_) with corresponding the area under the curve (*A*_FA_) as *A*_FA _= (1.1 × 10^8^) *V*_FA _+ 4.045. This suggests that CO_2_ reduction and hydrogen evolution processes take place simultaneously at the cathode, as described by the proposed mechanism. In both the reactions, protons (H^+^) are required and more H^+^ was utilized towards H_2_ evolution reaction.

### Current density near electrodes

**Figure 6:**
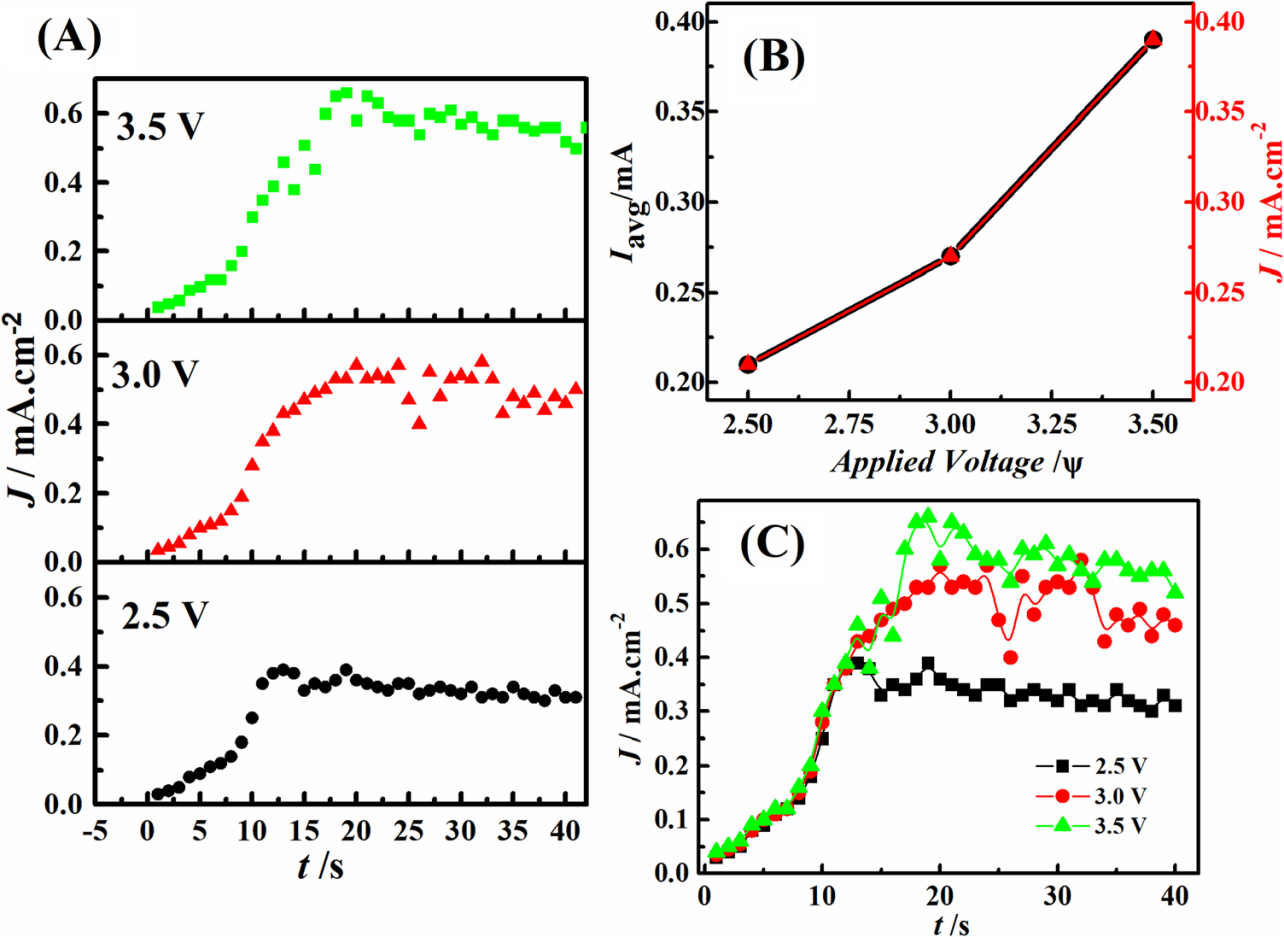
(**A**) The plot represents the variation in current density (*J*) with time (*t*) in seconds (s), when the electric potential (*ψ*) was varied from 2.5 V, 3.0 V, to 3.5 V, respectively. The image (**B**) shows variations of the average current (*I*_*avg*_—black squares) and average current density (*J*_*avg*_—red circles) with applied voltage, and the image (**C**) shows the variation of current density (J) with time (t) collected together when the electric potential (*ψ*) was varied from 2.5 V, 3.0 V, to 3.5 V.

The Fig. 6A represents that the current density (*J*) in the microreactor increased with time (*t*) = 0–15 s before reaching a saturation value after *t *= 15–40 s, at the discrete electric potential or voltage (*ψ*) values of 2.5 V, 3.0 V, and 3.5 V, while a constant flowrate of 3 mL/min is maintained for the seawater and CO_2_ within the microchannel. At (*ψ*) = 3.0 V, the irregularities around *t *= 20–30 s, can be attributed to the reduction of gaseous CO_2_ and H_2_ gas evolution, occurring simultaneously at the cathode. The experiments were performed independent of each other at various voltage values. The Fig. 6B shows that the average current (*I*_*avg*_—left *y* axis) and average current density (*J*_*avg*_—right *y* axis) of the microfluidic reactor increased linearly with the applied electric potential (*ψ*). It must be noted that since the distance between the two electrodes was close to unity the two curves almost overlap each other. An increment in current density signifies higher reaction rate along with H_2_ gas evolution. The Fig. 6C shows a very similar trend as Fig. 6A for all the electric potential (*ψ*) values ranging from 2.5 V to 3.5 V when the readings are recorded in a single experiment by ramping up the voltage across the electrodes. In the microreactor, the system involves the formation of organic products at the downstream, and for error-free calculation of current density, the distance between the two electrodes was kept constant for all the experiments.

### Microfluidic device prototype

**Figure 7:**
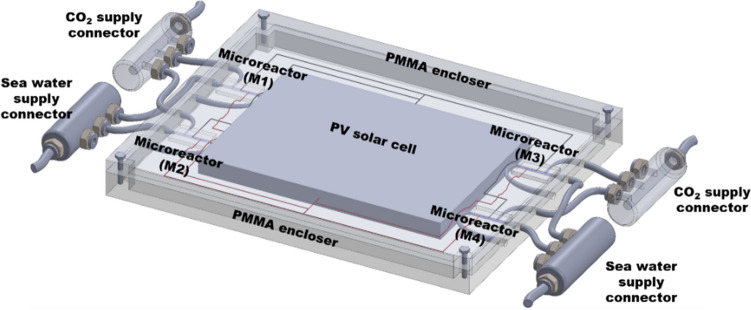
The schematic represents an envisioned microfluidic device consisting of four microreactor units, an integrated PV solar cell module, CO_2_ gas and seawater inlets through connectors with their corresponding electronic circuitry^44^.

We propose a microfluidic device (Patent number: 332899) enclosed inside a poly (methyl methacrylate) (PMMA) or acrylic glass. The Fig. 7 illustrates an envisioned membraneless microreactor-based device^44^ which can be employed for the production of diverse organic species like formic acid, aldehydes, alcohols, formate salts, hydrocarbons, aliphatic and aromatic esters, primary and secondary alcohols using solar energy and in absence of any catalyst. The gaseous CO_2_ and the salty seawater can be introduced through inlets, respectively of the microchannels embedded in the microreactor. The proposed microfluidic device will comprise of an integrated PV solar panel, four microreactor units (M1-M4), and electrical connections, all encased within the PMMA framework. This ergonomic design makes this solar energy-driven microfluidic device more versatile and efficient for synthesis of value-added organic compounds relative to the PDMS template-based microreactor proposed in this study.

## Discussion

In summary, we designed a membraneless PDMS-based microreactor prototype comprising of built-in Cu electrodes linked to an external photovoltaic solar panel, and a T-shaped microchannel using the template molding technique for sequestration of greenhouse CO_2_ gas. The naturally abundant CO_2_ gas was introduced in to the microreactor and was converted into various value-added organic compounds using seawater as electrolyte and solar irradiation as energy source, thereby, emulating the photosynthesis process. As the integrated solar panel efficiently converts the sunlight into the electrical energy and the Cu electrodes, which are separated by a microscale distance, create a high-intensity electric field at a lower potential difference for the co-electrolysis of CO_2_ and seawater, the proposed microreactor required a low operating electrical power for chemical reactions. The incoming gaseous CO_2_ and seawater after flowing into the region of high intensity electric field, undergoes rapid co-electrolysis in absence of any catalyst, produces nascent hydrogen, nascent oxygen and free radicals which in turn participates in the synthesis of diverse organic products ranging from aldehyde, formate salts, formic acid, primary or secondary alcohols, to short-chain hydrocarbons within the microreactor under continuous solar illumination. Importantly, by controlling the electric field intensity produced by the solar cells under solar irradiation, the reaction rate, and the amount of organic compound to be synthesized were regulated and optimized for industrial applications. The proposed proof-of-concept microfluidic reactor opens up the avenue for CO_2_ sequestration and production organic compounds as it uses greenhouse CO_2_ gas as reactant to synthesize a wide range of organic products. Furthermore, in near future, a very large-scale integration (µVLSI) of the these microreactors might help in scaling-up the production of diverse organic compounds in larger volumes.

## Methods

### Filtration of seawater

The seawater was vacuum filtered by passing it through ultrafine Nylon 66 Filter Membrane using an oil-free vacuum pump for removal of undesirable solid contaminants, and the filtered seawater was used as electrolyte in the experiments. The pH and electrical conductivity of seawater after filtration was measured to be ~ 7.78 and ~4231 µS cm^−1^, at room temperature, respectively. The filtered seawater was used in all the experiments, unless stated otherwise.

### Co-electrolysis of seawater and CO_2_ under natural solar irradiation

The template molding technique was used to fabricate a microreactor consisting of a two inlet-shaped microchannel with a diameter of ~ 500 μm and the copper (Cu) electrodes mounted in a position perpendicular to the microchannel. The detailed fabrication protocol of the microreactor is shown in the Fig. S1 in ESI of the manuscript. The PV panels were attached to the Cu electrodes and the solar panels were held for 2 h under solar light to stabilize the output voltages before starting the experiments. The solar panels were integrated directly inside the microchannel in the absence of any inverter with a power tolerance in the range of ± 3%. Thereafter, the output voltage was measured with the digital multimeter for finalizing the output voltage for performing the experiments.

The electric field was varied by partly covering the solar panel with the aid of a black sheet. The applied electrical field potentials finalized for the tests were 2.5 V, 3 V and 3.5 V. In some of the experiments, solar PV panels were incorporated in series in order to verify the intensity of the applied electrical field. Seawater was injected into one of the microreactor inlets at a steady flow rate by a syringe pump (Harvard Apparatus, PHD 2000) and gaseous CO_2_ was inserted into one of the inlets by maintaining a continuous supply of CO_2_ from a commercial CO_2_ gas cylinder. The gas-liquid mixture of CO_2_ and seawater was flown through the microreactor to generate different organic chemicals under solar illumination. For each experiment, 5 ml of sea water was injected at a flow rate of *Q*_*w *_= 3 mL/min and the CO_2_ gas was introduced at flow rates of *Q*_*g *_= 3 mL/min into the microchannel. Importantly, by controlling the field strength across the electrodes, the rate of organic compound synthesis were varied efficiently and the reaction intermediates and the organic products were collected downstream in a closed vial for further analysis.

## Supplementary Information

Supplementary Information.

## Data Availability

The datasets used and/or analyzed during the current study is available from the corresponding author on request.
