# Supplementary material for: Co-electrolysis of seawater and carbon dioxide inside a microfluidic reactor to synthesize speciality organics
Source: Sci Rep. 2023 Jun 26;13:10298. doi: 10.1038/s41598-023-34456-6 (PMC10293243; doi:10.1038/s41598-023-34456-6)
Supplement: Supplementary file 1 — Supplementary Information. [file 41598_2023_34456_MOESM1_ESM.docx]

**Electronic Supplementary Information**

**Co-electrolysis of Seawater and Carbon Dioxide inside a Microfluidic Reactor to Synthesize Speciality Organics**

Saptak Rarotra,^1‡^ Amit Kumar Singh,^2#*^ Tapas Kumar Mandal,^1,2^ and Dipankar Bandyopadhyay ^1,2*^

*^1^Department of Chemical Engineering, Indian Institute of Technology Guwahati, Assam - 781039, India.*

*^2^Centre for Nanotechnology, Indian Institute of Technology Guwahati, Assam - 781039, India.*

*‡Current address: Energy Research Institute, Nanyang Technological University, Singapore - 637553, Singapore.*

*# Current address: Department of Mechanical Engineering, George Mason University, Virginia – 22030, USA.*

*Email address: [asingh91@gmu.edu](mailto:asingh91@gmu.edu) (A.K.S.); [dipban@iitg.ac.in](mailto:dipban@iitg.ac.in) (D. B.)

Tel: +91-361-2582254; Fax: +91-361-2582291

**1. Materials**

Sodium chloride (NaCl), acetone (C_2_H_6_O), sulfuric acid (H_2_SO_4_) (98%), hydrogen peroxide (H_2_O_2_) (50%), Potassium bromide (KBr), and ethanol (C_2_H_5_OH) (99.9%) were obtained from Merck (India). Single stranded copper (Cu) microwires of diameter ~500 µm were procured from Surgeon Sons, India. Tetrabutyl ammonium hydrogen sulfate (C_16_H_37_NO_4_S) and Nylon 66 Filter Membranes of pore size 0.45 μm were procured from Sigma Aldrich (India). The Borosilicate glass membrane filter holder assembly were procured from Amazon (India). The gaseous CO_2_ (99.99% purity) was procured from Assam air products. Poly-dimethylsiloxane (PDMS) SYLGARD-184 kit was procured from Dow Corning, India (for microreactor fabrication. The aforementioned chemicals were of analytical grade and used without further purification. Milli-Q grade water was used for cleaning and solution preparation. The stainless-steel tailoring needles, glass slides, double-sided tape, and the Pidilite Fevikwik Adhesive instant glue were procured from the local market. The photovoltaic (PV) solar cell panels were purchased from Aditi Solar Pvt. Ltd., India, with maximum open and close circuit voltages of 10.1 V and 8.5 V, respectively. Seawater used in the experiments was collected from Bay of Bengal, Odisha, India.

**2. Characterization**

In order to examine the functional groups of the compounds found in the samples, Fourier transform infrared spectroscopy (FT-IR, Shimadzu, Japan, model: IRAffinity-1) was used. The end products (in liquid form) were collected downstream from the microreactor for FT-IR analysis. In the collected aqueous solution, organic products were present in colloidal form. To obtain a powder sample, the solution was completely vacuum-dried. This results in a powder that contains many organic compounds formed during the reaction. In order to obtain pellets for FT-IR analysis, the powder sample was mixed with KBr at a fixed weight ratio (sample: KBr = 1:100). At room temperature, FT-IR studies were conducted on all samples in transmission mode. An FT-IR study of the pellets yields a single spectrum containing peaks corresponding to different or similar functional groups present in various organic compounds.

Furthermore, the reaction intermediates were also analyzed by a gas chromatograph (GC, Varian, 450-GC, Netherlands) connected to a mass spectrometer (MS, Varian, 240-MS, Netherlands). During the analysis, 1 μL concentrated solution was injected to GC-MS instrument. In the GC, the injector was operated at 260°C with the split closed for 5 min. Further, helium (>99.999% pure) was used as the carrier gas at a flow rate of 1 mL/min. The VF-5 ms (Varian, Netherlands) column having dimension of 30 m × 0.25 mm was used for analysis. The thickness of the stationary phase column film was 0.25 μm. The initial column oven temperature was maintained at 60°C for 4 min, and then the temperature was ramped up to 150°C at a rate of 5°C/min. Following this, the temperature was raised from 150°C to 280°C at a rate of 15°C/min and then the temperature was then maintained at 280°C for 5 min. For MS investigations, the temperature of the ion source was set at 300°C and the detector voltage was maintained at 1 kV for analysis in the electron impact (70 eV) ionization mode. The data were acquired in the full-scan detection mode ranging from 35 to 350 a.m.u. at a rate of 0.5 scan/s. Products were identified by comparing the mass spectra with NIST-2010 Standard Reference Database.

The characterization of H_2_ and O_2_ gases were performed by GC instrument. The temperature of the injector, oven, and detector were fixed to 100°C while nitrogen (N_2_) carrier gas was maintained at a flowrate of 10 mL/min during analysis and 5 ml of analyte were injected for the sample analysis. In order to quantify the amount of H_2_ and O_2_ gas produced by the microreactor, a calibration curve of pure H_2_ and O_2_ gases was initially prepared using GC analysis [9]. A calibration curve for pure H_2_ and O_2_ gases were obtained by injecting different volumes of pure H_2_ and O_2_ gases (0.1 ml – 1 ml) into the TCD port of the GC using a Hamilton 100 μL gastight syringe.

The products from CO_2_ reduction were analyzed by ultra-fast liquid chromatography. (UFLC, Shimadzu LC-20AD, UV-detector of deuterium lamp SPD-20A). The product was detected at 205 nm wavelengths by injecting 20 μL of reacting sample to the C-18 Column (10 × 4 mm). For this analysis, 5 mM tetrabutyl ammonium hydrogen sulfate was used as mobile phase at flow rate of 1 mL/min. The dimension of the microreactor were measured using a Leica DM 2500 upright microscope. The output voltage was measured with a MASTECH M92A (H) digital multimeter with a measuring DC voltage range of 200 mV -1000 V DC and accuracy level of ± (0.5% +3). The pH and the electrical conductivity of seawater was measured with a CyberScan Scan pH 510 meter (Eutech Instruments) and handheld HM digital AquaPro Water Quality Tester, respectively.

**3. Fabrication of Membraneless Microreactor**


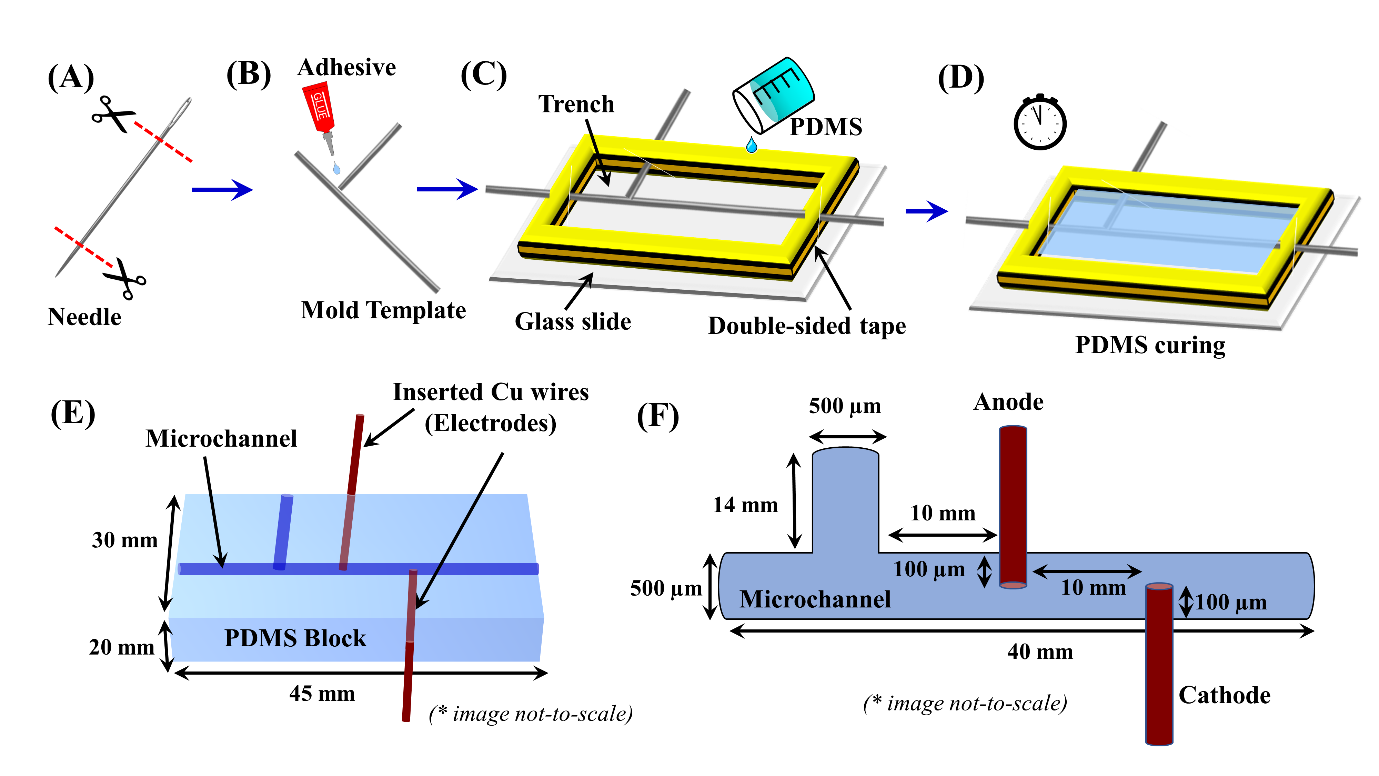


**Fig. S1.** Schematic illustration depicting the steps for the fabrication of microreactor.

The microreactor was fabricated by the template molding technique using PDMS substrate and commercial stainless-steel tailoring needles of ~500 μm diameter as template (**Fig. S1)**. Initially, two needles were cut at both ends to form hollow rods (**Fig. S1A)** and then needles were glued with each other to form a T-shaped molding template (**Fig. S1B)** with excellent tensile strength and smooth outer surface finish. A rectangular trench was subsequently created on a glass slide using double-sided tapes (**Fig. S1C)**. Following this, the template was cleaned with ethanol and acetone and then it was positioned on the trench in a desired orientation. The PDMS precursor and the curing agent solution were mixed in a 10:1 (v/v) ratio, degassed to remove trapped air bubbles, and then the uncured mixture was poured in the rectangular trench, and cured in an oven at 65 °C for 40 min to form a solid PDMS block (**Fig. S1D)**. After the PDMS was cured, the trench structure was disassembled and the rectangular PDMS block (45 mm × 30 mm × 20 mm) was obtained by detaching the cured PDMS from the glass slide. Following this, the PDMS block was dipped in acetone to swell the cured polymer. The embedded needle template was manually pulled out from the PDMS block, leaving behind a smooth T-shaped microchannel. The microchannel typically had the diameter of ~500 μm, as verified under optical microscope. The microchannel was cleaned by ultra-sonication in acetone and methanol baths for 10 min followed by treatment with 10% (v/v) piranha solution (H_2_SO_4_:H_2_O_2_, 3:1) for 15 min and air-dried. The Cu microwires were inserted in the perpendicular direction to the microchannel at the center to form electrodes (**Fig. S1F)**. The **Fig. S1E** and **Fig. S1F** displays the typical dimension and geometry of the microreactor along with the positioning details of the Cu electrodes.

**4. Parameters for GC-MS Data Analysis**

| **Parameters** | **Values** |
| --- | --- |
| Lock Peak Width | Yes |
| Parameters: | Local Set |
| Peak Width (sec): | 4.0 |
| Slope Sensitivity (SN): | 10 |
| Tangent %: | 10 |
| Peak Size Reject (counts): | 2000 |
| Smoothing: | Mean 5 Point Smooth |

**Tables S1.** Details of GC-MS parameters with their corresponding values used for the analysis of the as obtained organics products after the completion of the electrochemical reaction inside the microreactor, operating at an applied potential (*ψ)* of 3.5 V. The flow rate of both sea water and CO_2_ was maintained at 3 mL/min.

**5. Calibration plot for H_2_ and O_2_ gas evolution**


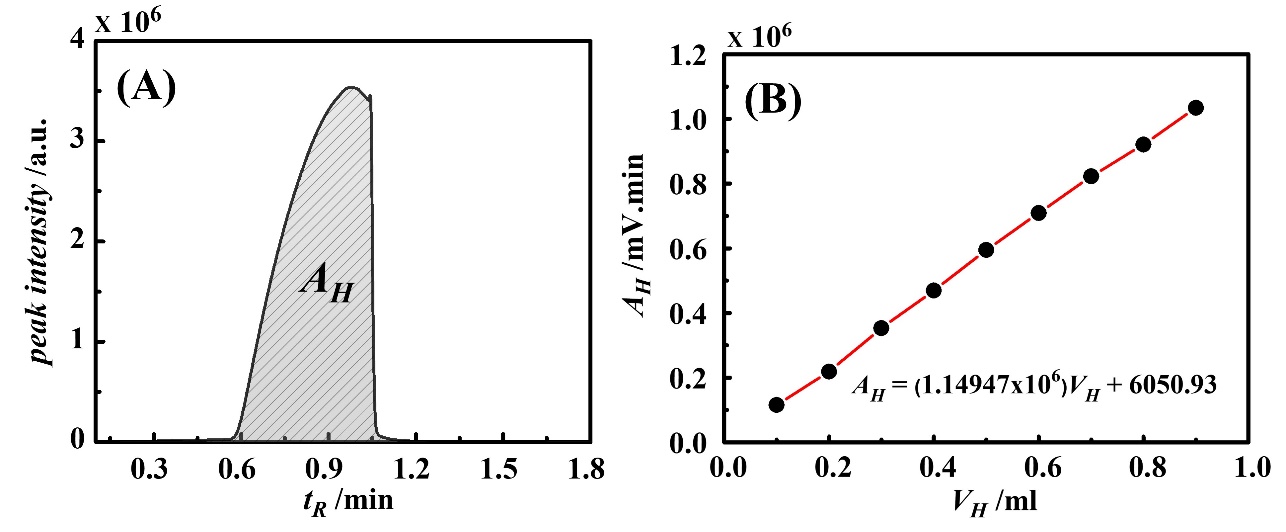


**Fig. S2.** **(A)** Pure hydrogen (H_2_) gas peak from the Gas Chromatography (GC). The peak at retention time (*t*_R_) in the range of ~ 0.6 -1.0 min represent H_2_ gas. The grey colored area inside the plot represents the area under the curve for the H_2_ (*A*_H_) gas **(B)** The calibration plot for H_2_ gas.

The **Fig. S2A** shows that for a particular volume of H_2_ gas, a GC peak with a well-defined area under the curve (*A*_H_) for the retention time in the range of 0.6 min – 1.0 min was obtained. A calibration curve for pure H_2_ gas was obtained by injecting different volumes of pure H_2_ gas (0.1 ml – 0.9 ml) into the TCD port of the GC. The **Fig. S2B** shows the linear correlation between the volume of pure hydrogen gas (*V*_H_) with corresponding area under the curve (*A*_H_) as, *A*_H_ = (1.14947 × 10^6^) *V*_H_ + 6050.93.


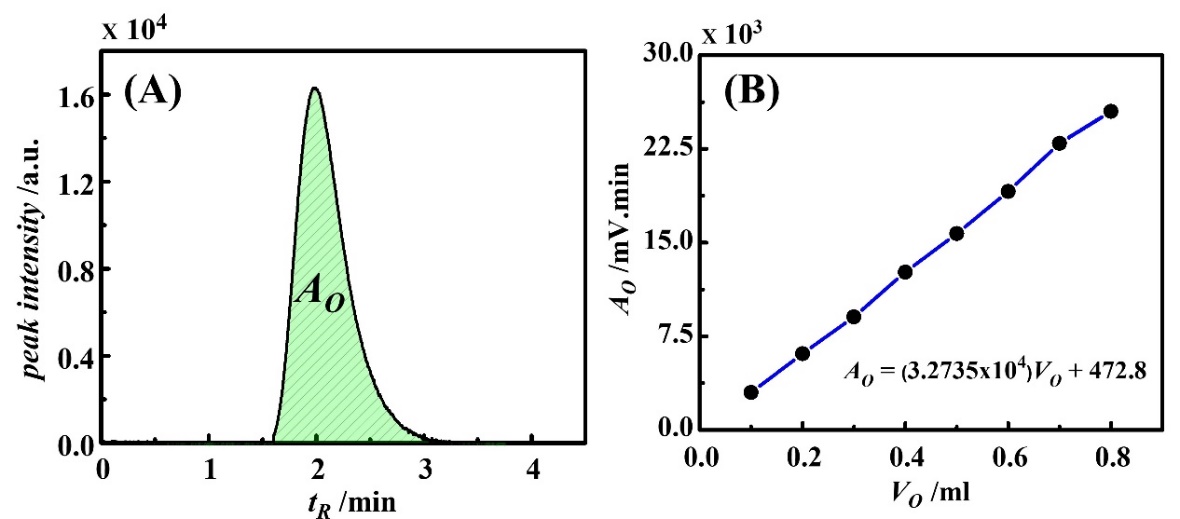


**Fig. S3.** **(A)** Pure hydrogen (O_2_) gas peak from the Gas Chromatography (GC). The peak at retention time (*t*_R_) in the range of ~1.5 – 2.0 min represent O_2_ gas. The grey colored area inside the plot represents the area under the curve for the O_2_ (*A*_O_) gas **(B)** The calibration plot for O_2_ gas.

The **Fig. S3A** shows the GC analysis of pure oxygen (O_2_) as reference gas. For a particular volume of O_2_ gas, a GC peak with a well-defined area under the curve (*A*o) for the retention time in the range of ~1.5 – 2.5 min was obtained. A calibration curve for pure O_2_ gas was obtained by injecting different volumes of pure O_2_ gas (0.1 ml – 0.8 ml) into the TCD port of the GC. The **Fig. S3B** shows the linear correlation between the volume of pure oxygen gas (*V*_O_) with corresponding area under the curve (*A*_O_) as, *A*o = (3.2735 × 10^4^) *V*o + 472.8.

**6. HPLC Analysis**


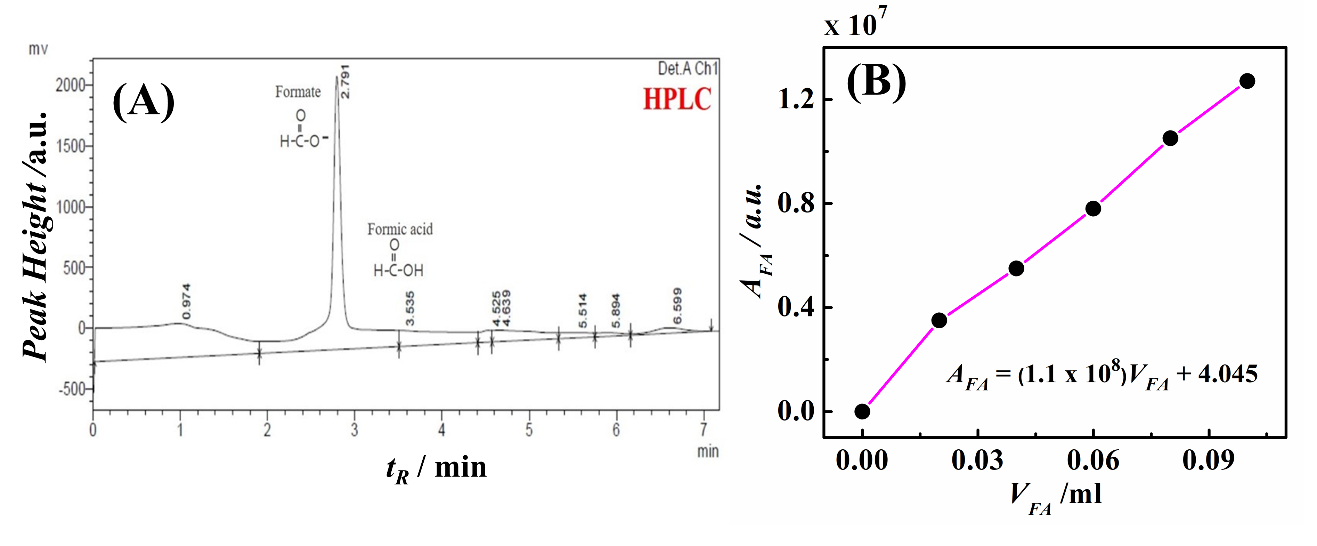


**Fig. S4.** The image **(A)** shows the HPLC spectrogram of organic products obtained from the microreactor outlet at *ψ* = 3.5 V, *Q*_w_ = 3 mL/min, and *Q*_g_ = 3 mL/min. The image **(B)** shows the calibration plot for the HPLC analysis of formic acid.

In the **Fig. S4A**, the peaks observed near the retention times of ~2.79 min and ~3.52 min corresponds to formate and formic acid species as obtained from the microreactor. The **Fig. S4B** shows the linear calibration plot between the volume of pure formic acid (*V*_FA_) with the area under the curve (*A*_FA_) as *A*_FA_ = (1.1 × 10^8^) *V*_FA_ + 4.045. A calibration curve for pure formic acid was obtained by injecting different volumes of 98-100% analytical grade formic acid (0.1 ml – 0.9 ml) into the HPLC instrument. A reacted solution of 20 mL was taken as source sample, which was injected through C-18, column at 205 nm wavelength.

**7. Current Density Calculations**

The Current Density (J) was calculated using the equation (1):

*J = I / A* ………………. (1)

where, J = Current density (in mA/cm^2^); A = The cross-section area of the microchannel (in cm^2^); I = Current flowing through the conductor (in mA), measured using multimeter.
